# Nasopharyngeal Carriage of Pneumococci Four Years after Community-Wide Vaccination with PCV-7 in The Gambia: Long-Term Evaluation of a Cluster Randomized Trial

**DOI:** 10.1371/journal.pone.0072198

**Published:** 2013-09-27

**Authors:** Anna Roca, Michel M. Dione, Abdoulie Bojang, John Townend, Uzochukwu Egere, Ousainou Darboe, Stephen R. C. Howie, Philip C. Hill, Richard A. Adegbola, Brian M. Greenwood, Martin Antonio

**Affiliations:** 1 Medical Research Council Unit, Banjul, The Gambia; 2 Centre for International Health, School of Medicine, University of Otago, Dunedin, New Zealand; 3 GlaxoSmithKline Vaccines, Wavre, Belgium; 4 Faculty of Infectious and Tropical Diseases, London School of Hygiene & Tropical Medicine, London, United Kingdom; Health Protection Agency, United Kingdom

## Abstract

**Background:**

A village-randomized trial of a seven-valent pneumococcal-conjugate-vaccine (PCV-7) conducted in rural Gambia showed a decrease of vaccine-type (VT) and a non-significant increase in non-vaccine-type (NVT) nasopharyngeal carriage of pneumococci two years after vaccination. Here, we report findings four years after vaccination.

**Methods:**

PCV-7 was given to all children below 30 months of age enrolled in the trial and to those born during its course in all study villages. Villages were randomized (older children and adults) to receive PCV-7 (wholly vaccinated villages) or serogroup-C-meningococcal-conjugate-vaccine (partly vaccinated villages). Cross-sectional surveys (CSS) to collect nasopharyngeal swabs were conducted before and at various intervals after vaccination. Sixteen of these randomized villages (8 wholly vaccinated and 8 partly vaccinated) participated in a CSS conducted four years after vaccination started.

**Results:**

Four years after vaccination, the prevalence of VT pneumococcal carriage was slightly higher in partly than in wholly vaccinated villages [6.4% versus 3.9% (p = 0.120)] compared to 24.4% in the pre-vaccination CSS (p<0.001). Prevalence of NVT four years after vaccination was similar between study groups [32.7% versus 29.8% (p = 0.392), respectively] compared to 51.1% in the pre-vaccination CSS (p<0.001). Four years after vaccination started, lower prevalence of serotype 6A was detected in wholly vaccinated than in partly vaccinated villages (1.6% versus 3.5%, p = 0.093) whilst the prevalence of serotype 19A was similar between groups (2.9% versus 2.5%, p = 0.779). The most prevalent serotype 19A clone was ST 847. The most prevalent serotype 6A clone before vaccination was ST3324 whilst after vaccination ST913 and ST1737 predominated. Fourteen out of 26 STs detected among the serotype 6A isolates were new while no new 19A serotype ST was found.

**Conclusions:**

The decline in prevalence of VT pneumococci seen shortly after PCV-7 vaccination was sustained four years later with only a small difference between study arms. No significant serotype replacement was detected.

**Trial Registration:**

ClinicalTrials.gov ISRCTN51695599

## Introduction

The prevention of pneumococcal disease is a major, international public health priority due to the high burden of disease and associated mortality, especially in children in developing countries [1]. The marked efficacy of pneumococcal conjugate vaccines (PCVs) in preventing pneumococcal disease from serotypes included in the vaccine (vaccine-serotypes or VT) occurs in large part through reduction of nasopharyngeal carriage [2], [3]. Because children are the major drivers of pneumococcal transmission [4], it is not surprising that after introduction of PCV vaccination in children, reduction in both nasopharyngeal carriage and invasive disease has been seen in older members of the vaccinated community [2], [5]–[9]. Some studies undertaken after the introduction of PCVs have shown an increase in both the incidence of invasive disease and the prevalence of carriage caused by serotypes not included in the vaccine (non-vaccine-serotypes or NVT), the phenomenon named serotype replacement [2], [3], [8], [10]–[13]. Serotype replacement has also been observed in non-vaccinated older members of vaccinated communities. However, the overall incidence of invasive pneumococcal disease has decreased following vaccination despite the emergence of serotype replacement [8].

It is likely that PCV vaccination facilitates serotype replacement by inducing a humoral and/or cellular immune response that makes it more difficult for VT pneumococci to colonize the nasopharynx, opening the way for colonization by NVT pneumococci and, perhaps, by other bacteria. In countries where PCV has been introduced into routine immunization programs, immune pressure against VT pneumococci will increase gradually as more vaccinated individuals enter the population. Determining the consequence of this would require long-term longitudinal studies. As an alternative, we had conducted a cluster randomized trial in which the pneumococcal population of some villages was exposed to maximum immune pressure by immunizing the whole population with PCV-7 (wholly vaccinated villages) with the primary objective of evaluating the effect on nasopharyngeal carriage of pneumococci compared with that seen in other villages where only children less than 30 months of age and newborns were vaccinated (partially vaccinated villages). The prevalence of carriage with VT pneumococci fell after vaccination in all age groups in both study arms, with a more marked drop in villages where the whole community had received PCV-7 [7]. There was little variation in the prevalence of NVT carriage following vaccine introduction [7].

In 2009, one year after the trial had finished, The Gambia introduced PCV-7 into the Expanded Programme on Immunization (EPI) and newborns in the study communities continued to receive 3 doses of PCV-7 given at 2,3 and 4 months of age. In October 2010, two years after the RCT had finished and 4 years after vaccination started, we conducted a further cross-sectional survey (CSS) to evaluate the long-term impact of PCV-7 vaccination on the prevalence of pneumococcal nasopharyngeal carriage in wholly and partially vaccinated villages. At the time of this survey, all children younger than 7 years of age had received PCV-7 in both study arms. The primary objectives of this long-term CSS were to evaluate the impact of PCV-7 on pneumococcal prevalence and serotype distribution in partly and wholly vaccinated villages four years after vaccine introduction. Additional objectives were to describe the population structure before and after vaccination of two key pneumococcal serotypes not present in PCV-7 that can be influenced by vaccination: i) serotype 6A, a serotype against which some cross protection might be seen following vaccination with PCV-7, which contains a serogroup 6B conjugate [14], and ii) serotype 19A, a serotype frequently associated with serotype replacement) [12], [13].

## Materials and Methods

### Study site

The study was carried out in the Sibanor area, Western Region, The Gambia. Twenty-one of the 55 villages in the study area were selected for inclusion in the initial trial with an overall population of 5441 in June 2006. Demographic characteristics of the study population have been described previously [15].

Study participants gave individual informed consent; parental consent was obtained for children up to 16 years of age. Both the initial trial and the long-term follow-up study were approved by the joint MRC/Gambia Government Ethics Committee and by the ethics committee of the London School of Hygiene & Tropical Medicine. Conduct of the trial was guided by a Data Safety and Monitoring Board [7].

### Trial design and surveys

The protocol for this trial and supporting CONSORT checklist are available as supporting information; see Checklist S1 and Protocol S1.

A village-randomized trial of the impact of PCV-7 on pneumococcal nasopharyngeal carriage was conducted in 21 Gambian villages between 2006 and 2008, with a baseline CSS conducted in 2003–2004 before vaccination started. Both vaccine recipients and laboratory personnel were blinded to village allocation.

Preparations for the study, study design, method of randomization and conduct of the CSS have been presented in detail previously [7]. In brief, because the non-licensed PCV-9 (which included serotypes 1 and 5 in addition to the PCV-7 serotypes) had already been shown to be effective in young children in a randomized controlled trial conducted in The Gambia trial [16], it was considered unethical not to give PCV-7 to young children in all the study villages. Therefore, all children in the trial aged between 2 and 30 months at enrolment, the age group covered by the PCV-9 trial [16], received PCV-7 as did any child born during the period of the study. Village randomization was applied to subjects above the age of 30 months (2.5 years of age) when vaccination started. On the basis of their randomization group, subjects above the age of 30 months received either one dose of PCV-7 (Prevenar®, Pfizer) (11 wholly vaccinated villages) or one dose of a meningococcal serogroup C conjugate vaccine (Meningitec®, Pfizer) (10 partly vaccinated villages). The meningococcal vaccine was given to ensure blinding and is not expected to have had any effect on pneumococcal carriage.

Only individuals 2.5 years of age and above participated in the CSS surveys. As part of the initial trial, CSS were conducted before vaccination (Baseline CSS) and 4–6, 12 and 22 months after vaccination (CSS-1 to CSS-3 respectively). During each CSS, nasopharyngeal swabs were collected from approximately 1,200 randomly selected, age stratified subjects [7]. Because of an azithromycin mass drug programme as part of the Gambian National Trachoma Elimination campaign prior to the CSS conducted 22 months after vaccination (CSS-3), only a smaller number of subjects could be included in this survey and this lead to a reduction in the mean age group. All samples included in the analysis of CSS-3 were collected before the azithromycin campaign started in any of the villages included in this survey. The main study ended in August 2008.

### Cross-sectional survey 4 (CSS-4)

One year after the trial finished (August 2009), The Gambia introduced PCV-7 into the EPI following the same schedule as that used for young infants in the trial (vaccination at 2, 3 and 4 months of age). In addition, older children up to 2 years of age were offered 1 dose of the vaccine.

Approximately 50 months after trial vaccination started and 15 months after national immunization with PCV-7 commenced, a follow-up CSS survey was conducted in 16 of the 21 original study villages: four villages were excluded as a consequence of participation in the azithromycin mass treatment campaign (3 months before the CSS started) and the fifth village, the smallest in the initial trial, declined to participate in the new survey. A nasopharyngeal swab (NPS) was collected from 800 age-stratified, randomly selected subjects during a one-month period (September–October 2010). Because the last update of the census had been conducted two years before, all individuals who subsequently moved into the study area were not included in the randomization and, as in the previous surveys, children younger than 2 years of age were not included as all had received PCV-7 [7].

### Sample handling

Sample handing and laboratory methods were similar in all the CSS surveys. NPS were collected, using a calcium alginate swab, from the posterior wall of the nasopharynx and immediately inoculated into vials containing skim milk-tryptone-glucose-glycerol (STGG) transport medium. Vials were placed in a cold box before transfer to the Medical Research Council Laboratories at Fajara within eight hours of collection and vials were stored at −70°C prior to analysis [17].

### Laboratory methods

#### Microbiology

10 µL of thawed, inoculated STGG medium were plated onto a gentamicin blood agar plate and incubated for 18–24 hours at 35°C in 5% CO_2_. Pneumococcal identification was based on colony morphology and conventional methods of characterization (optochin susceptibility and bile solubility assays) [15]. Serotyping was performed at the MRC Fajara Laboratories, with capsular and factor typing sera (Statens Serum Institut, Copenhagen, Denmark), using a modified latex agglutination assay [18]. Equivocal results were confirmed by the Quellung reaction.

#### Penicillin susceptibility

Serotype 6A and 19A isolates were first screened using oxacillin disk diffusion. Oxacillin resistant isolates that could be recovered from storage were subsequently tested for penicillin susceptibility using E-test strips.

#### PCR

For all 6A/C serotypes detected by latex agglutination, a PCR with specific primers for 6C was used to differentiate 6C isolates from 6A [19], [20].

#### Multilocus Sequence Typing (MLST)

MLST was performed on a restricted random selection of isolates of serotypes 6A (n = 55; 1/3 of this number from the pre-vaccination survey and 2/3 from the post-vaccination surveys) and 19A (n = 43; 1/3 of this number from the pre-vaccination survey and 2/3 from the post-vaccination surveys)Samples from CSS-3 were not available. Seven housekeeping genes were targeted: *aroE*, *gdh*, *gki*, *recP*, *spi*, *xpt* and *ddl*. Amplification of all genes and separation of fragments was carried out as described elsewhere [21].

### Data management and statistical analysis

Pneumococcal serotypes were grouped as follows: (i) VT: serotypes included in PCV-7 (4, 6B, 9V, 14, 18C, 19F, and 23F) plus 6A which shows cross-protective immunity with serotype 6B; and (ii) NVT: any other pneumococcal serotype including non-typeable pneumococci. Individuals carrying both a VT and an NVT at the same time were counted in both groups. The primary outcomes of the study for each of the age groups were (i) the prevalence of carriage of VT pneumococci and (ii) the prevalence of carriage of NVT pneumococci.

Individuals were age grouped following widely used but arbitrary age categories as follows: (i) young children: individuals 2.5 to less than 5 years of age; (ii) older children: individuals 5 years to less than 15 years of age; and (iii) adults: individuals aged 15 years and above. We excluded children less than 2.5 years as they had been vaccinated in both study arms. Because children up to 7 years had been vaccinated in both arms in CSS-4, we compared groups in this last CSS considering also an age subgroup - children 7 years to less than 15 years.

The sample size for the last study CSS was calculated so as to be able to detect a 60% reduction in the prevalence of VT pneumococcal carriage in each age group between wholly vaccinated and partly vaccinated villages, with 80% power at a two-sided 5% significance level [from 25% to 10% in the younger and older children (n = 100 per arm and age group), and from 15% to 6% in adults (n = 200 per arm)]. Whilst the study was not powered to detect effects on individual serotypes we carried out exploratory analyses on serotypes 6A and 19A to look for possible effects on these important serotypes and these are presented alongside the main analyses. Given the relatively low power of the study to detect effects on individual serotypes, observations relating to 6A and 19A should be interpreted with caution.

Comparisons of quantitative measures were made using Wilcoxon rank-sum tests. Comparisons of categorical variables were made using Chi-square tests. For each of the surveys, comparison of VT and NVT pneumococcal carriage between study arms was done using mixed effects logistic regression with village included as a random effect to allow for the cluster randomized design. Mixed effects logistic regression was used to compare carriage in different surveys within each study arm. The analyses were carried out using Stata 11 (StataCorp); *p*-values less than 0.05 were taken to indicate statistical significance.

Sequences were edited and complementary sense antisense fragments were aligned using the Laser Gene DNA star 7.1 software. The sequences were submitted to the MLST database website (www.mlst.net) and assigned to existing or novel allele or sequence type numbers defined by the database. Sequence types (STs) were analyzed for relatedness using Bionumerics software (version 6.5; Applied Maths, Sint-Martens-Latem, Belgium).

## Results

### Study profile

**Figure 1:**
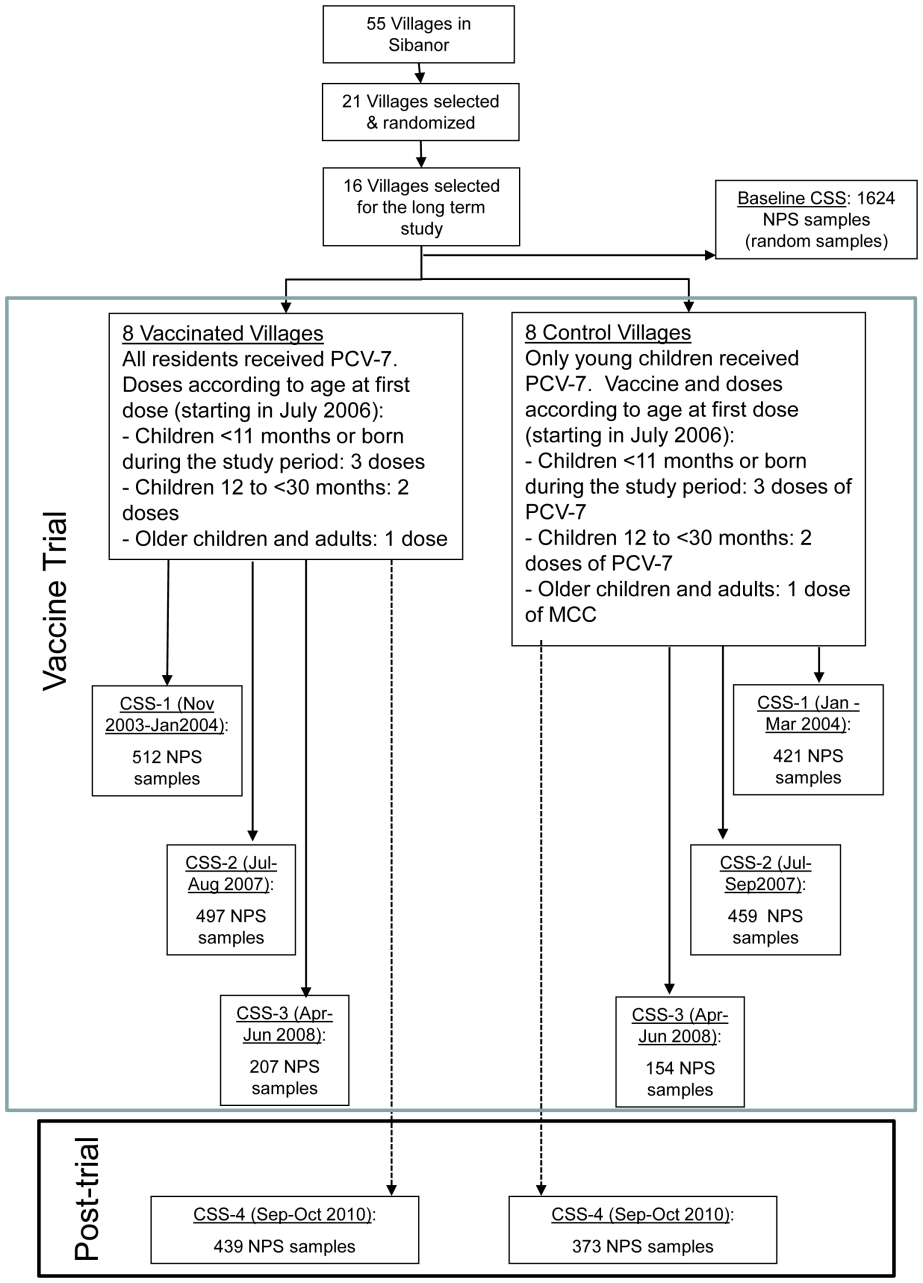
Trial profile. PCV-7: 7-valent pneumococcal conjugate vaccine; CSS-1: Cross-sectional 1; CSS-2: Cross-sectional 2; CSS-3: Cross-sectional 3; CSS-4: Cross-sectional 4.

Sixteen villages participated in the long-term survey (8 wholly vaccinated and 8 partly vaccinated); 1624 NPS samples were collected from residents of these 16 villages in the pre-vaccination survey, 933, 956 and 361 NPS samples in the first three post-vaccination surveys and 812 NPS samples in the long-term post-vaccination survey reported in this paper (Figure 1). Table 1 provides a summary of the median age and sex of individuals included in each of the surveys. Because of an azithromycin mass drug programme prior to CSS-3 only a reduced sample of subjects could be included in this survey and this lead to a reduction in the mean age group.

**Table 1:** Age and sex distribution of participants in five CSS undertaken in partly and wholly vaccinated villages.

CSS survey	Variable		Partly vaccinated %(n)	Wholly vaccinated %(n)	p-value
**Baseline**	Age group				0.160
	(years)	2.5-<5	11.1 (77)	9.9(92)	
		5-<15	32.6(227)	37.1(344)	
		> = 15	56.4(393)	53.0(491)	
	Median Age (years)		16.5	15.7	0.315
	Sex	Male	51.6 (360)	45.6(423)	0.016
**CSS-1**	Age group				0.242
	(years)	2.5-<5	11.4 (48)	14.6(75)	
		5-<15	38.5 (162)	34.6(177)	
		> = 15	50.1 (211)	50.8(260)	
	Median Age (years)		15.3	15.5	0.911
	Sex	Male	48.9(206)	45.1(231)	0.245
**CSS-2**	Age group				0.799
	(years)	2.5-<5	12.0(55)	12.7(63)	
		5-<15	37.0(170)	35.0(174)	
		> = 15	51.0(234)	52.3(260)	
	Median Age (years)		15.5	16.3	0.641
	Sex	Male	47.3(217)	48.9(243)	0.617
**CSS-3**	Age group				0.242
	(years)	2.5-<5	20.1(31)	13.5(28)	
		5-<15	50.6(78)	55.6(115)	
		> = 15	29.2(45)	30.9(64)	
	Median Age (years)		8.5	8.6	0.212
	Sex	Male	48.0(74)	48.3(100)	0.961
**CSS-4**	Age group				0.696
	(years)	2.5-<5	23.6(88)	25.5(112)	
		5-<15	27.6(103)	25.3(111)	
		> = 15	48.8(182)	49.2(216)	
	Median Age (years)		14.0	14.7	0.662
	Sex	Male	50.9(190)	51.7(227)	0.827

### Prevalence of pneumococcal carriage in the long-term follow-up survey

Overall prevalence of pneumococcal carriage in CSS-4 was 35.5% (288/812), decreasing with increasing age (67.0% in young children, 39.3% in older children and 17.6% in adults). Prevalence of VT pneumococcal carriage was 5.0% (41/812) (11.0% among young children, 7.9% among older children and 0.5% among adults). The most common VT isolated was serotype 6A (n = 20), representing 2.5% of all NPS samples; followed by serotypes 14 (n = 8) and 19F (n = 7). The overall prevalence of NVT carriage was 31.2% (253/812) and also decreased with age (58.0% among young children, 31.8% among older children and 17.3% among adults). Approximately 10% of serotypes included in the NVT group were non-typeable (n = 24). Serotype 19A (n = 22) was the most prevalent NVT serotype, representing 2.7% of all NPS samples, followed by serotypes 21 (n = 18) and serotype 35B (n = 17). Serotype 6C isolates were not detected.

### Comparisons of wholly vaccinated versus partly vaccinated villages

Prevalence of any pneumococcal carriage and NVT carriage during CSS-4 was similar in wholly vaccinated and partly vaccinated villages, except for a lower prevalence of any carriage in vaccinated villages among older children (31% versus 47.6%, p = 0.036). The prevalence of carriage of VT pneumococci was lower, although not statistically significantly so, also among the older children group (5–15 year old) resident in wholly vaccinated villages compared to partly vaccinated villages (5.4% versus 10.7%, p = 0.161) (Table 2). This difference became more remarkable, although still not statistically significant, when only children aged 7-<15 years old were considered, excluding those children in the partially vaccinated villages aged 5-<7 years old who may have been vaccinated. In children in 7-<15 year old age group resident in fully or partly vaccinated villages the prevalence of pneumococcal carriage of any serotypes was 26.8% versus 46.2% (OR = 0.43, p = 0.015) for all pneumococci, 1.4% versus 9.0% (OR = 0.14, p = 0.074) for VT pneumococci and 25.4% versus 38.5% (OR = 0.54, p = 0.089) for NVT pneumococci.

**Table 2:** Prevalence of nasopharyngeal carriage of any pneumococcus (Any type), vaccine serotypes (VT), non-vaccine serotypes (NVT) and serotypes 6A and 19A detected during CSS-4 (four years after vaccination) in partly and wholly vaccinated villages by age group.

		Partly vaccinated		Wholly vaccinated				
Serotype Group	Age group (years)	%	(n/N)	%	(n/N)	OR (95% CI)		p-value
**Any Type**	2.5-<5	67.0%	(59/88)	67.0%	(75/112)	1.12	(0.47, 2.66)	0.803
	5-<15	47.6%	(49/103)	31.5%	(35/111)	0.47	(0.23, 0.95)	0.036
	> = 15	19.2%	(35/182)	16.2%	(35/216)	0.81	(0.48, 1.36)	0.430
	All ages	38.3%	(143/373)	33.0%	(145/439)	0.79	(0.54, 1.16)	0.233
**VT**	2.5-<5	13.6%	(12/88)	8.9%	(10/112)	0.69	(0.20, 2.32)	0.547
	5-<15	10.7%	(11/103)	5.4%	(6/111)	0.48	(0.17, 1.34)	0.161
	> = 15	0.5%	(1/182)	0.5%	(1/216)	0.84	(0.05,13.56)	0.903
	All ages	6.4%	(24/373)	3.9%	(17/439)	0.59	(0.31, 1.15)	0.120
**NVT**	2.5-<5	55.7%	(49/88)	59.8%	(67/112)	1.19	(0.67, 2.09)	0.556
	5-<15	37.9%	(39/103)	26.1%	(29/111)	0.56	(0.28, 1.10)	0.094
	> = 15	18.7%	(34/182)	16.2%	(35/216)	0.84	(0.50, 1.42)	0.516
	All ages	32.7%	(122/373)	29.8%	(131/439)	0.87	(0.64, 1.19)	0.392
**Serotype 6A (VT)**	2.5-<5	6.8%	(6/88)	3.6%	(4/112)	0.51	(0.14, 1.85)	0.304
	5-<15	5.8%	(6/103)	2.7%	(3/111)	0.45	(0.11, 1.84)	0.267
	> = 15	0.5%	(1/182)	0.0%	(0/216)	0.00	------------	0.998*
	All ages	3.5%	(13/373)	1.6%	(7/439)	0.45	(0.18, 1.14)	0.093
**Serotype 19A (NVT)**	2.5-<5	8.0%	(7/88)	4.5%	(5/112)	0.55	(0.16, 1.85)	0.332
	5-<15	1.9%	(2/103)	5.4%	(6/111)	2.74	(0.29,25.76)	0.378
	> = 15	1.1%	(2/182)	0%	(0/216)	0.00	-------------	0.457*
	All ages	2.9%	(11/373)	2.5%	(11/439)	0.86	(0.30, 2.48)	0.779

* p-values obtained using Fisher's exact test.

The overall prevalence of serotype 6A was higher in partly vaccinated versus wholly vaccinated villages (3.5% versus 1.6%, p = 0.093). As a result, if we exclude serotype 6A in the VT goups, the difference between wholly vaccinated and partly vaccinated villages increases. The prevalence of serotype 19A was similar in both groups of villages (2.9% versus 2.5%, p = 0.779) [Table 2].

### Time trends in the prevalence of pneumococcal carriage

**Figure 2:**
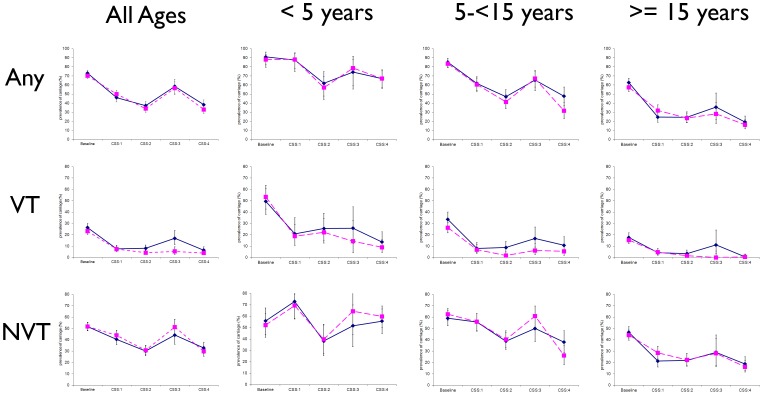
Prevalence of pneumococcal carriage of any serotype (Any), vaccine serotype (VT), and non-vaccine serotype (NVT) in cross-sectional surveys before and after vaccination with PCV-7 stratified by age group and study arm (solid lines indicate partly vaccinated villages and dashed lines indicate wholly vaccinated villages).

**Figure 3:**
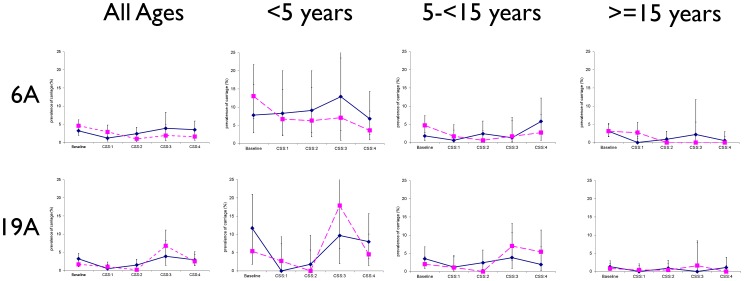
Prevalence of pneumococcal carriage of serotypes 6A (VT group) and 19A (NVT group) in cross-sectional surveys before and after vaccination with PCV-7 stratified by age group and study arm (solid lines indicate partly vaccinated villages and dashed lines indicate wholly vaccinated villages).

In partly vaccinated villages, prevalence of pneumococcal carriage was significantly lower in CSS-4 compared to the baseline CSS for overall pneumococcal carriage and VT carriage (all age groups) and for NVT carriage (older children and adults). For some serotypes there was a lower prevalence of carriage in CSS-4 compared to previous post-vaccination surveys (CSS-1 to 3) [Figure 2, Table 3 and Table 4]. For serotype 6A, prevalence in CSS-4 was lower than in the pre-vaccination survey (significantly so for young children and adults p = 0.019 and p = 0.008 respectively). Serotype 19A prevalence was similar over the whole observation period with a transitory increase in the wholly vaccinated study arm in CSS-3, mostly driven by the youngest age group that returned to the initial rate in CSS-4 (Figure 3, Table 3 and Table 4).

**Table 3:** Comparison of prevalence of nasopharyngeal carriage of any pneumococcal carriage (Any type), vaccine serotypes (VT), non-vaccine serotypes (NVT) and serotypes 6A and 19A in different cross-sectional surveys in partly vaccinated villages.

		CSS-4 vs Baseline		CSS-4 vs CSS-1		CSS-4 vs CSS-2		CSS-4 vs CSS-3	
Serotype group	Age Group (years)	OR (95%CI)	p-value	OR (95%CI)	p-value	OR (95%CI)	p-value	OR (95%CI)	p-value
**Any type**	2.5-<5	0.18 (0.07,0.46)	<0.001	0.27 (0.10,0.71)	0.008	1.26 (0.61,2.60)	0.538	0.69 (0.25,1.89)	0.467
	5-<15	0.15 (0.09,0.25)	<0.001	0.53 (0.32,0.88)	0.014	1.02 (0.62,1.67)	0.950	0.52 (0.28,0.97)	0.040
	> = 15	0.154 (0.10,0.24)	<0.001	0.80 (0.49,1.31)	0.373	0.80 (0.50,1.30)	0.367	0.49 (0.24,1.02)	0.056
	All ages	0.20 (0.15,0.26)	<0.001	0.76 (0.56,1.02)	0.071	1.11 (0.82,1.49)	0.494	0.60 (0.40,0.89)	0.011
**VT**	2.5-<5	0.15 (0.07,0.33)	<0.001	0.58 (0.23,1.48)	0.253	0.42 (0.18,1.01)	0.052	0.35 (0.12,1.03)	0.057
	5-<15	0.21 (0.10,0.42)	<0.001	1.27 (0.54,2.99)	0.577	1.21 (0.53,2.76)	0.657	0.68 (0.28,1.63)	0.388
	> = 15	0.02 (0.003,0.18)	<0.001	0.12 (0.01,0.96)	0.046	0.15 (0.02,1.23)	0.077	0.04 (0.01,0.37)	0.004
	All ages	0.18 (0.11,0.28)	<0.001	0.85 (0.49,1.47)	0.558	0.79 (0.46,1.35)	0.383	0.42 (0.23,0.76)	0.004
**NVT**	2.5-<5	1.02 (0.55,1.89)	0.955	0.47 (0.22,1.01)	0.054	2.19 (1.09,4.40)	0.028	1.43 (0.60,3.41)	0.413
	5-<15	0.42 (0.26,0.67)	<0.001	0.48 (0.29,0.80)	0.004	0.96 (0.58,1.59)	0.869	0.63 (0.35,1.14)	0.129
	> = 15	0.29 (0.19,0.44)	<0.001	0.94 (0.57,1.56)	0.825	0.90 (0.55,1.48)	0.692	0.67 (0.31,1.43)	0.302
	All ages	0.44 (0.33,0.58)	<0.001	0.75 (0.55,1.01)	0.055	1.18 (0.87,1.60)	0.274	0.78 (0.52,1.15)	0.211
**Serotype 6A (VT)**	2.5-<5	0.79 (0.24,2.62)	0.702	0.72 (0.19,2.77)	0.638	0.63 (0.18,2.23)	0.474	0.34 (0.08,1.47)	0.148
	5-<15	3.61 (0.98,13.34)	0.054	9.86 (1.16,83.63)	0.036	2.59 (0.71,9.49)	0.150	4.85 (0.56,41.7)	0.151
	> = 15	0.16 (0.02),1.29)	0.085	Infinite (---)^a^	0.463	0.60 (0.05,6.73)	0.678	0.21 (0.01,3.59)	0.283
	All ages	1.10 (0.54, 2.22)	0.792	3.09 (1.09,8.76)	0.034	1.50 (0.66,3.40)	0.332	1.09 (0.40,2.95)	0.860
**Serotype 19A (NVT)**	2.5-<5	0.62 (0.22,1.80)	0.381	Infinite (---)^a^	0.051	4.28 (0.50,36.42)	0.183	0.58 (0.12,2.78)	0.499
	5-<15	0.51 (0.11,2.46)	0.402	1.48 (0.20,10.73)	0.697	0.80 (0.14,4.48)	0.803	0.56 (0.09,3.45)	0.531
	> = 15	1.03 (0.19,5.48)	0.968	Infinite (---)^a^	0.214	1.53 (0.21,11.13)	0.673	Infinite (---)^a^	1.000
	All ages	0.83 (0.39,1.77)	0.637	6.22 (1.36,28.45)	0.018	1.90 (0.72,5.01)	0.192	0.99 (0.35,2.75)	0.983

a It was not possible to fit the model.

OR's were calculated ignoring clustering and p-values using Fisher's exact test.

**Table 4:** Comparison of prevalence of nasopharyngeal carriage of any pneumococcal carriage (Any type), vaccine serotypes (VT), non-vaccine serotypes (NVT) and serotypes 6A and 19A in different cross-sectional surveys in wholly vaccinated villages.

Serotype group	Age group (years)	CSS-4 vs Baseline		CSS-4 vs CSS-1		CSS-4 vs CSS-2		CSS-4 vs CSS-3	
		OR (95%CI)	p-value	OR 95%CI	p-value	OR (95%CI)	p-value	OR (95%CI)	p-value
**Any**	2.5-<5	0.28 (0.13,0.58)	0.001	0.27 (0.12,0.61)	0.002	1.58 (0.83,3.01)	0.165	0.49 (0.18,1.37)	0.173
	5-<15	0.07 (0.04,0.12)	<0.001	0.25 (0.15,0.41)	<0.001	0.55 (0.33,0.93)	0.024	0.21 (0.12,0.38)	<0.001
	> = 15	0.16 (0.11,0.24)	<0.001	0.46 (0.29,0.73)	0.001	0.75 (0.47,1.20)	0.225	0.56 (0.29,1.09)	0.088
	All ages	0.17 (0.13,0.22)	<0.001	0.46 (0.35,0.61)	<0.001	0.99 (0.74,1.32)	0.924	0.45 (0.31,0.64)	<0.001
**VT**	2.5-<5	0.08 (0.04,0.18)	<0.001	0.42 (0.18,1.02)	0.057	0.35 (0.14,0.85)	0.021	0.49(0.14,1.78)	0.280
	5-<15	0.14 (0.06,0.33)	<0.001	0.70 (0.25,1.93)	0.489	2.80 (0.68,11.48)	0.154	0.91(0.29,2.81)	0.870
	> = 15	0.03 (0.00,0.15)	<0.001	0.10 (0.00,0.66)	0.008	0.30 (0.01,3.04)	0.383	Inf (---)^a^	1.000
	All ages	0.13 (0.07,0.21)	<0.001	0.49 (0.27,0.89)	0.019	0.90 (0.47,1.73)	0.75	0.84 (0.38,1.82)	0.653
**NVT**	2.5-<5	1.37 (0.78,2.38)	0.272	0.66 (0.35,1.22)	0.184	2.27 (1.21,4.26)	0.011	0.81 (0.33,1.95)	0.638
	5-<15	0.19 (0.12,0.31)	<0.001	0.25 (0.15,0.43)	<0.001	0.48 (0.2830.82)	0.007	0.22 (0.12,0.39)	<0.001
	> = 15	0.28 (0.19,0.42)	<0.001	0.55 (0.35,0.87)	0.010	0.80 (0.49,1.28)	0.346	0.56 (0.29,1.09)	0.087
	All ages	0.38 (0.29,0.49)	<0.001	0.53 (0.40,0.70)	<0.001	0.98 (0.73,1.31)	0.908	0.47 (0.33,0.67)	<0.001
**Serotype 6A (VT)**	2.5-<5	0.25 (0.08,0.80)	0.019	0.51 (0.13,1.97)	0.330	0.55 (0.13,2.28)	0.410	0.43 (0.07,2.59)	0.359
	5-<15	0.51 (0.14,1.82)	0.300	1.49 (0.29,7.63)	0.635	4.54 (0.46,45.05)	0.196	1.51 (0.24,9.37)	0.656
	> = 15	0.00 (0.00,0.57)	0.008	0.00 (0.00,0.65)	0.018	-b	-	-b	-
	All ages	0.32 (0.14,0.72)	0.006	0.53 (0.21,1.31)	0.170	1.57 (0.49,4.99)	0.447	0.94 (0.27,3.27)	0.927
**Serotype 19A (NVT)**	2.5-<5	0.81 (0.22,2.90)	0.742	1.62 (0.30,8.65)	0.574	Infinite (---)^a^	0.161	0.16 (0.04,0.67)	0.013
	5-<15	2.37 (0.76,7.32)	0.135	4.40 (0.86,22.42)	0.074	Infinite (---)^a^	0.003	0.79 (0.26,2.37)	0.677
	> = 15	0.00 (0.00,2.18)	0.319	0 (0., ---)	1.000	0 (0, ---)	1.000	0 (0, ---)	0.229
	All ages	1.28 (0.58,2.82)	0.542	2.393 (0.82,6.99)	0.111	11.50 (1.47,89.83)	0.020	0.41 (0.18,0.93)	0.033

a It was not possible to fit the model. OR's were calculated ignoring clustering and p-values using Fisher's exact test.

b Values could not be calculated.

Overall, the percentage of serotype 6A among VT isolates increased over the study surveys (16.4% in the baseline CSS; 28.6%, 27.6% and 27.0% in CSS-1 to CSS-3 respectively; and 48.8% in CSS-4 –p<0.001). At the same time, the prevalence of serotype 19A among NVT isolates also varied over the study surveys (4.5% in the baseline CSS,1.8%, 2.8% and 11.5% in CSS-1 to CSS-3; and 8.7% in CSS-4 – p<0.001).

### Penicillin susceptibility for serotypes 6A and 19A isolates

Penicillin susceptibility was evaluated in 126/131 serotype 6A isolates and all but one were susceptible. This isolate, which was obtained in the baseline survey, had an intermediate level of resistance. For serotype 19A, 90/96 isolates were tested five of which were non-susceptible (intermediate level of resistance); one of these isolates was obtained in the baseline survey and the remaining four in CSS-4 (2 in the wholly vaccinated communities and 2 in the partly vaccinated communities). The prevalence of non-susceptibility among 19A isolates in CSS-4 was 18.2% (4/22).

### Molecular analysis of serotypes 6A and 19A isolates

#### Serotype 6A genotyping

One hundred and thirty-one serotype 6A isolates were obtained over the course of the five surveys. Genotyping analysis was performed on 55 (42.0%) (16/65 from the pre-vaccination survey, 26/36 from CSS-1 and CSS-2; and 13/20 from CSS-4). Twenty-six STs were found, among which 14 were new with one isolate each. The most prevalent clone before vaccination was ST3324; after vaccination the most prevalent clone was ST913 followed by ST1737, representing respectively 10.9%, 14.5% and 12.7% of the analysed isolates (Table 5). Only two of 14 new STs were found before vaccination, the rest were detected only after vaccination. Most of the new genotypes clustered around ST1737 which was seen before and after vaccination. ST919, ST3324 and ST1737 were more commonly found in vaccinated than in control villages.

**Table 5:** Distribution of Sequence Types (ST) of 55 serotype 6A pneumococcal isolates randomly selected from the different CSS surveys.

ST	Allelic profile							Study CSS				
	*Aroe*	*gdh*	*gki*	*recP*	*spi*	*xpt*	*ddl*	Baseline	CSS-1	CSS-2	CSS-4	Total
913	6	57	83	28	7	19	9	2	2	0	4	8
1737	6	57	34	28	6	1	9	2	2	2	1	7
3324	7	8	1	5	42	60	14	5	0	0	1	6
919	16	38	4	16	7	358	28	2	1	1	0	4
5734	7	25	29	2	27	122	122	1	1	1	1	4
1288	60	82	1	4	42	122	28	0	0	2	1	3
4036	16	214	4	18	15	1	31	1	1	0	0	2
5716	16	214	4	18	15	4	31	1	0	0	1	2
8045*	7	57	83	28	7	19	14	0	1	1	0	2
912	6	57	34	28	7	1	9	0	1	0	0	1
3407	23	189	4	12	43	4	74	0	0	0	1	1
5527	1	25	4	4	6	122	28	0	0	0	1	1
5702	2	8	9	10	17	83	6	0	0	1	0	1
8039*	7	57	83	28	7	19	9	1	0	0	0	1
8040*	6	57	29	2	6	112	9	1	0	0	0	1
8041*	7	57	29	1	27	1	74	0	1	0	0	1
8042*	6	57	29	28	7	1	9	0	1	0	0	1
8043*	6	8	1	28	7	16	14	0	1	0	0	1
8044*	6	57	4	28	6	358	28	0	1	0	0	1
8046*	6	57	34	5	6	1	9	0	1	0	0	1
8047*	6	10	29	1	7	122	74	0	1	0	0	1
8048*	60	82	1	1	42	21	28	0	0	1	0	1
8049*	2	8	9	10	17	3	6	0	0	1	0	1
8050*	54	5	4	4	2	142	269	0	0	1	0	1
8053*	8	5	36	3	7	1	6	0	0	0	1	1
8054*	2	8	4	41	15	4	31	0	0	0	1	1
**Total**								**16**	**15**	**11**	**13**	**55**

* New Sequence Types described in this study. For the 13 new ST in the post-vaccination period, 7 come from samples collected in vaccinated villages and 6 from control villages (2 of them from PCV-7 vaccinated children).

#### Serotype 19A genotyping

Ninety-six serotype 19A isolates were obtained over the course of the five surveys. Forty-three were randomly selected for genotyping (15/38 from the pre-vaccination survey; 15/15 from CSS-1 and CSS-2; and 12/22 from CSS-4). Fourteen STs of serotype 19A were described among these 43 isolates and none was new. ST847 was the most prevalent during both the pre and post vaccination period, representing 58.1% (n = 25) of all analyzed isolates (Table 6). All STs were equally distributed between vaccinated and control villages except for ST4029 which was more commonly found in control villages.

**Table 6:** Distribution of Sequence Types (ST) of 43 serotype 19A pneumococcal isolates randomly selected from the different CSS surveys.

	Allelic profile							Study CSS				
ST	*aroE*	*gdh*	*gki*	*recP*	*spi*	*xpt*	*ddl*	Baseline	CSS-1	CSS-2	CSS-4	Total
847	7	11	4	1	6	112	14	8	6	2	9	25
4029	13	5	62	18	9	1	19	1	1	0	1	3
2174	7	16	8	8	6	142	14	2	0	0	0	2
4033	118	8	4	12	17	19	6	2	0	0	0	2
7324	7	11	4	5	6	112	14	0	0	2	0	2
4101	7	11	4	1	72	112	14	0	1	0	0	1
4102	1	215	53	2	10	28	18	1	0	0	0	1
4107	7	11	4	1	6	28	14	1	0	0	0	1
4109	13	5	62	18	9	1	19	1	0	0	0	1
5732	8	11	4	1	6	112	14	0	0	1	0	1
7321	5	6	1	47	6	1	29	0	0	0	1	1
7334	11	5	4	10	17	1	8	0	0	1	0	1
7337	15	17	4	42	6	20	161	0	0	0	1	1
7339	54	2	4	4	2	142	269	0	0	1	0	1
**Total**								**16**	**8**	**7**	**12**	**43**

## Discussion

To anticipate the potential long-term effects of the introduction of PCV in sub-Saharan Africa, we conducted a novel CRT in which several villages in rural Africa were exposed to maximum immune pressure by vaccinating all residents with PCV-7 and compared the prevalence of pneumococcal carriage in these villages with that in villages where only children less than 30 months had been vaccinated [7]. The initial drop in the prevalence of VT pneumococcal nasopharyngeal carriage observed in all age groups early after vaccination in both wholly vaccinated and partly vaccinated villages was attributed to the herd effect of vaccine introduction in young children, whilst the slightly greater drop observed in wholly vaccinated than in partly vaccinated villages was attributed to the direct effect of the vaccine.

Our analysis shows that more than four years after PCV-7 vaccination the overall prevalence of pneumococci was similar in fully and partly vaccinated villages with a trend towards lower prevalence among young children (5-<15 years age group) in the wholly vaccinated communities. For the same age group, the prevalence of VT pneumococcal carriage was lower in fully vaccinated compared to partly vaccinated villages (5.4% versus 10.7%), although this difference was not statistically significant (p = 0.161). However, in a sub-analysis, not defined in the original statistical plan, undertaken in children aged 7-<15 years to exclude any vaccinated children resident in the partly vaccinated villages, the difference between groups became more remarkable (1.4% versus 9.0%) with borderline statistical significance (p = 0.074) suggesting that vaccination of a whole village had provided some benefit to these older children. However, this later finding needs to be treated with caution as it is based on an analysis undertaken after the initial results had been obtained and p-values had not been adjusted by the number of groups compared. The time trend analysis showed a continuous decrease of VT pneumococcal carriage and no increase in NVT prevalence in both groups of villages. In contrast to what has been observed in some other areas [22], VT have not been eliminated yet from the study area and several more years of vaccination may be needed to achieve this goal.

Serotype 6A, not included in PCV-7 but with potential cross-protection from 6B polysaccharide and thus included in the VT grouping, was the most prevalent VT. Although the study lacked power for individual serotypes comparison, prevalence of serotype 6A was a lower (not statistically significant), in wholly vaccinated villages. The time-trend analysis showed that the decrease of this serotype after vaccination was slower in partly vaccinated villages than for other VT, suggesting that cross-protection of serotype 6A with serotype 6B was incomplete. This finding is in agreement with the observation of a smaller decline in the incidence of invasive pneumococcal disease caused by serotype 6A pneumococci compared to serotype 6B pneumococci following PCV-7 vaccination [23] and by the fact that PCV-7 can generate functional 6A antibody but only for two thirds of the samples tested [24]. In our study, serotype 6A was three times more prevalent among VT isolates in CSS-4 compared to the baseline survey. Genotyping of serotype 6A isolates showed high clonal dissemination of 6A genotypes both before and after vaccination with most new genotypes being encountered after vaccine introduction. No serotype 6C isolate was detected and so far, serotype 6C does not seem to have a strong presence in the region. If we would have excluded serotype 6A from the VT group analysis, differences between study arms in CSS-4 would have increased.

Prevalence of NVT was similar in wholly vaccinated and partly vaccinated villages more than 4 years after vaccination, indicating that replacement has not been favored by the increased vaccine pressure in wholly versus partly vaccinated villages. No increase in the overall prevalence of NVT carriage was observed in either study group during CSS-4 when compared to the baseline CSS, indeed there was a decrease in some age groups. This was an unexpected finding to which we do not have any satisfactory explanation. However, it is a finding which is robust as the methodologies used for sample collection, storage and laboratory testing were similar between the pre-vaccination and post-vaccination surveys and quality controls were in place throughout the study. It cannot be attributed to any health intervention by the study field team which had left the study villages more than two years prior to conducting CSS-4. The mass azithromycin treatment campaigns undertaken in some study villages more than two years before the last survey and in some surrounding villages 3–4 months before CSS-4 was conducted might have altered the transmission patterns in the area, as mingling between the populations of different villages does occur but this seems unlikely. Furthermore, azithromycin mass treatment campaigns tend to have a short term effect on nasopharyngeal carriage of pneumococci [25], [26]. No other changes in antibiotic policy occurred during the study period. Thus, we are left with the assumption there has been some secular trend in the pattern of pneumococcal transmission occurring during the course of the study that was independent of vaccination which we have not been able to characterise. Data from Upper River Region, The Gambia has also shown a trend for a decrease in pneumococcal carriage in children unrelated to vaccination [27]. This observed decrease in both VT and NVT led to a decrease in the overall prevalence of pneumococcal carriage in the study area.

A reassuring finding was that the overall prevalence of serotype 19A, an important cause of serotype replacement in many geographical areas [2], [3], [10], [13], [28], did not increase following vaccination, although there was an increase in the proportion of NVT isolates due to this serotype, and the prevalence of carriage with this serotype was no higher in wholly vaccinated than in partly vaccinated villages. In addition, genotyping data showed that all genotype 19A clustered with the presumed founder ST847 which was the most prevalent throughout the study, suggesting a stable population. It has been speculated that serotype 19A replacement is driven by antibiotic selection pressure [10], although recent data from Norway have shown that replacement of serotype 19A, at least in the case of invasive disease, can occur in the absence of penicillin resistance [28]. In our study, high level penicillin resistance was not detected although a higher prevalence of intermediate resistance was observed in the last study CSS. Sustained monitoring of this serotype is warranted as proportionally it had become the most prevalent of all NVT isolates during CSS-4.

A community trial conducted over a period of 7 years faces challenges, some of which could have influenced the findings reported in this paper. As discussed above, socio-economic changes may have happened in the community over this long time period which have not been recognized by the investigators. Furthermore, a potential impact of seasonality on pneumococcal carriage was not considered during the design of the initial study. However, CSS-4 was undertaken at the end of the rainy season as was the case for the initial baseline study.

Despite the challenges considered above, we have shown that the decrease of VT carriage among Gambian villagers observed shortly after vaccination continued for a further four years and the herd effect resulting from vaccination of young children was sustained. However, there is a trend towers lower prevalence of VT prevalence as a result of vaccinating older children and adults. Furthermore the prevalence of NVT, including that of serotype 19A, has not increased. A few months after the completion of CSS-4, PCV-13, which contains conjugates representing most of the current nasopharyngeal carriage prevalent serotypes in the study area, replaced PCV-7 in the Gambian EPI programme. The effect of this new vaccine on nasopharyngeal carriage of pneumococci in these communities will be monitored.

## Supporting Information

Checklist S1 **CONSORT Checklist.**
(DOC)

Protocol S1 **Trial protocol.**
(PDF)

## References

[1] O'Brien KL, Wolfson LJ, Watt JP, Henkle E, Deloria-Knoll M, et al (2009) Burden of disease caused by Streptococcus pneumoniae in children younger than 5 years: global estimates. Lancet 374: 893–902 S0140-6736(09)61204-6 [pii];10.1016/S0140-6736(09)61204-6 [doi] 19748398 10.1016/S0140-6736(09)61204-6

[2] Cheung YB, Zaman SM, Nsekpong ED, Van Beneden CA, Adegbola RA, et al (2009) Nasopharyngeal carriage of Streptococcus pneumoniae in Gambian children who participated in a 9-valent pneumococcal conjugate vaccine trial and in their younger siblings. Pediatr Infect Dis J 28: 990–995 10.1097/INF.0b013e3181a78185 [doi] 19536041 10.1097/INF.0b013e3181a78185

[3] Dagan R, Givon-Lavi N, Zamir O, Sikuler-Cohen M, Guy L, et al (2002) Reduction of nasopharyngeal carriage of Streptococcus pneumoniae after administration of a 9-valent pneumococcal conjugate vaccine to toddlers attending day care centers. J Infect Dis 185: 927–936 JID010101 [pii];10.1086/339525 [doi] 11920317 10.1086/339525

[4] Hill PC, Townend J, Antonio M, Akisanya B, Ebruke C, et al (2010) Transmission of Streptococcus pneumoniae in rural Gambian villages: a longitudinal study. Clin Infect Dis 50: 1468–1476 10.1086/652443 [doi] 20420503 10.1086/652443

[5] Lexau CA, Lynfield R, Danila R, Pilishvili T, Facklam R, et al (2005) Changing epidemiology of invasive pneumococcal disease among older adults in the era of pediatric pneumococcal conjugate vaccine. JAMA 294: 2043–2051 294/16/2043 [pii];10.1001/jama.294.16.2043 [doi] 16249418 10.1001/jama.294.16.2043

[6] Pilishvili T, Lexau C, Farley MM, Hadler J, Harrison LH, et al (2010) Sustained reductions in invasive pneumococcal disease in the era of conjugate vaccine. J Infect Dis 201: 32–41 10.1086/648593 [doi] 19947881 10.1086/648593

[7] Roca A, Hill PC, Townend J, Egere U, Antonio M, et al (2011) Effects of Community-Wide Vaccination with PCV-7 on Pneumococcal Nasopharyngeal Carriage in The Gambia: A Cluster-Randomized Trial. PLoS Med 8: e1001107 10.1371/journal.pmed.1001107 [doi];PMEDICINE-D-11-00302 [pii] 22028630 10.1371/journal.pmed.1001107 PMC3196470

[8] Whitney CG, Farley MM, Hadler J, Harrison LH, Bennett NM, et al (2003) Decline in invasive pneumococcal disease after the introduction of protein-polysaccharide conjugate vaccine. N Engl J Med 348: 1737–1746 10.1056/NEJMoa022823 [doi];348/18/1737 [pii] 12724479 10.1056/NEJMoa022823

[9] Whitney CG, Pilishvili T, Farley MM, Schaffner W, Craig AS, et al (2006) Effectiveness of seven-valent pneumococcal conjugate vaccine against invasive pneumococcal disease: a matched case-control study. Lancet 368: 1495–1502 S0140-6736(06)69637-2 [pii];10.1016/S0140-6736(06)69637-2 [doi] 17071283 10.1016/S0140-6736(06)69637-2

[10] Hanage WP, Finkelstein JA, Huang SS, Pelton SI, Stevenson AE, et al (2010) Evidence that pneumococcal serotype replacement in Massachusetts following conjugate vaccination is now complete. Epidemics 2: 80–84 10.1016/j.epidem.2010.03.005 [doi] 21031138 10.1016/j.epidem.2010.03.005 PMC2963072

[11] Hanquet G, Kissling E, Fenoll A, George R, Lepoutre A, et al (2010) Pneumococcal serotypes in children in 4 European countries. Emerg Infect Dis 16: 1428–1439. 20735928 10.3201/eid1609.100102 PMC3294971

[12] Hsieh YC, Lin PY, Chiu CH, Huang YC, Chang KY, et al (2009) National survey of invasive pneumococcal diseases in Taiwan under partial PCV7 vaccination in 2007: emergence of serotype 19A with high invasive potential. Vaccine 27: 5513–5518 S0264-410X(09)00981-5 [pii];10.1016/j.vaccine.2009.06.091 [doi] 19615960 10.1016/j.vaccine.2009.06.091

[13] van Gils EJ, Veenhoven RH, Hak E, Rodenburg GD, Keijzers WC, et al (2010) Pneumococcal conjugate vaccination and nasopharyngeal acquisition of pneumococcal serotype 19A strains. JAMA 304: 1099–1106 304/10/1099 [pii];10.1001/jama.2010.1290 [doi] 20823436 10.1001/jama.2010.1290

[14] Saeland E, Jakobsen H, Ingolfsdottir G, Sigurdardottir ST, Jonsdottir I (2001) Serum samples from infants vaccinated with a pneumococcal conjugate vaccine, PncT, protect mice against invasive infection caused by Streptococcus pneumoniae serotypes 6A and 6B. J Infect Dis 183: 253–260 JID000888 [pii];10.1086/317934 [doi] 11110649 10.1086/317934

[15] Hill PC, Akisanya A, Sankareh K, Cheung YB, Saaka M, et al (2006) Nasopharyngeal carriage of Streptococcus pneumoniae in Gambian villagers. Clin Infect Dis 43: 673–679 CID39492 [pii];10.1086/506941 [doi] 16912937 10.1086/506941

[16] Cutts FT, Zaman SM, Enwere G, Jaffar S, Levine OS, et al (2005) Efficacy of nine-valent pneumococcal conjugate vaccine against pneumonia and invasive pneumococcal disease in The Gambia: randomised, double-blind, placebo-controlled trial. Lancet 365: 1139–1146 S0140-6736(05)71876-6 [pii];10.1016/S0140-6736(05)71876-6 [doi] 15794968 10.1016/S0140-6736(05)71876-6

[17] O'Brien KL, Nohynek H (2003) Report from a WHO Working Group: standard method for detecting upper respiratory carriage of Streptococcus pneumoniae. Pediatr Infect Dis J 22: e1–11 10.1097/01.inf.0000049347.42983.77 [doi] 10.1097/01.inf.0000049347.42983.77 12586987

[18] Brueggemann AB, Pai R, Crook DW, Beall B (2007) Vaccine escape recombinants emerge after pneumococcal vaccination in the United States. PLoS Pathog 3: e168 07-PLPA-RA-0363 [pii];10.1371/journal.ppat.0030168 [doi] 18020702 10.1371/journal.ppat.0030168 PMC2077903

[19] Dias CA, Teixeira LM, Carvalho MG, Beall B (2007) Sequential multiplex PCR for determining capsular serotypes of pneumococci recovered from Brazilian children. J Med Microbiol 56: 1185–1188 56/9/1185 [pii];10.1099/jmm.0.47347-0 [doi] 17761481 10.1099/jmm.0.47347-0

[20] Pai R, Gertz RE, Beall B (2006) Sequential multiplex PCR approach for determining capsular serotypes of Streptococcus pneumoniae isolates. J Clin Microbiol 44: 124–131 44/1/124 [pii];10.1128/JCM.44.1.124-131.2006 [doi] 16390959 10.1128/JCM.44.1.124-131.2006 PMC1351965

[21] Antonio M, Dada-Adegbola H, Biney E, Awine T, O'Callaghan J, et al (2008) Molecular epidemiology of pneumococci obtained from Gambian children aged 2–29 months with invasive pneumococcal disease during a trial of a 9-valent pneumococcal conjugate vaccine. BMC Infect Dis 8: 81 1471-2334-8-81 [pii];10.1186/1471-2334-8-81 [doi] 18547404 10.1186/1471-2334-8-81 PMC2440749

[22] Scott JR, Millar EV, Lipsitch M, Moulton LH, Weatherholtz R, et al (2012) Impact of more than a decade of pneumococcal conjugate vaccine use on carriage and invasive potential in Native American communities. J Infect Dis 205: 280–288 jir730 [pii];10.1093/infdis/jir730 [doi] 22128315 10.1093/infdis/jir730 PMC3244367

[23] Hicks LA, Harrison LH, Flannery B, Hadler JL, Schaffner W, et al (2007) Incidence of pneumococcal disease due to non-pneumococcal conjugate vaccine (PCV7) serotypes in the United States during the era of widespread PCV7 vaccination, 1998–2004. J Infect Dis 196: 1346–1354 JID38151 [pii];10.1086/521626 [doi] 17922399 10.1086/521626

[24] Cooper D, Yu X, Sidhu M, Nahm MH, Fernsten P, et al (2011) The 13-valent pneumococcal conjugate vaccine (PCV13) elicits cross-functional opsonophagocytic killing responses in humans to Streptococcus pneumoniae serotypes 6C and 7A. Vaccine 29: 7207–7211 S0264-410X(11)00920-0 [pii];10.1016/j.vaccine.2011.06.056 [doi] 21689707 10.1016/j.vaccine.2011.06.056 PMC3170457

[25] Fry AM, Jha HC, Lietman TM, Chaudhary JS, Bhatta RC, et al (2002) Adverse and beneficial secondary effects of mass treatment with azithromycin to eliminate blindness due to trachoma in Nepal. Clin Infect Dis 35: 395–402 CID020038 [pii];10.1086/341414 [doi] 12145722 10.1086/341414

[26] Leach AJ, Shelby-James TM, Mayo M, Gratten M, Laming AC, et al (1997) A prospective study of the impact of community-based azithromycin treatment of trachoma on carriage and resistance of Streptococcus pneumoniae. Clin Infect Dis 24: 356–362. 9114185 10.1093/clinids/24.3.356

[27] Akinsola AK, Ota MO, Enwere GC, Okoko BJ, Zaman SM, et al (2012) Pneumococcal antibody concentrations and carriage of pneumococci more than 3 years after infant immunization with a pneumococcal conjugate vaccine. PLoS One 7: e31050 10.1371/journal.pone.0031050 [doi];PONE-D-11-14054 [pii] 22363544 10.1371/journal.pone.0031050 PMC3282700

[28] Vestrheim DF, Steinbakk M, Aaberge IS, Caugant DA (2012) Postvaccination increase in serotype 19A pneumococcal disease in Norway is driven by expansion of penicillin-susceptible strains of the ST199 complex. Clin Vaccine Immunol 19: 443–445 CVI.05563-11 [pii];10.1128/CVI.05563-11 [doi] 22237889 10.1128/CVI.05563-11 PMC3294600

